# How Should We Test for Lynch Syndrome? A Review of Current Guidelines and Future Strategies

**DOI:** 10.3390/cancers13030406

**Published:** 2021-01-22

**Authors:** Richard Gallon, Peter Gawthorpe, Rachel L. Phelps, Christine Hayes, Gillian M. Borthwick, Mauro Santibanez-Koref, Michael S. Jackson, John Burn

**Affiliations:** Faculty of Medical Sciences, Newcastle University, Newcastle upon Tyne NE1 3BZ, UK; p.gawthorpe2@newcastle.ac.uk (P.G.); rachel.phelps@newcastle.ac.uk (R.L.P.); christine.hayes@newcastle.ac.uk (C.H.); gillian.borthwick@newcastle.ac.uk (G.M.B.); mauro.santibanez-koref@newcastle.ac.uk (M.S.-K.); michael.jackson@newcastle.ac.uk (M.S.J.)

**Keywords:** Lynch syndrome, screening, mismatch repair deficiency

## Abstract

**Simple Summary:**

Carriers of Lynch syndrome (LS) have an increased risk for several types of tumour, in particular bowel and uterine cancers. LS is caused by inheritance of a faulty mismatch repair (MMR) gene, and, in accordance with clinical guidelines, bowel cancers are tested for MMR deficiency to screen for LS. We review the significant barriers to following these guidelines in routine practice, and discuss guideline limitations. We also look at the advances in our knowledge and technology that may address some of these barriers and limitations. We further discuss additional LS screening strategies, in particular MMR deficiency testing of other tumour types and normal tissues to identify LS gene carriers.

**Abstract:**

International guidelines for the diagnosis of Lynch syndrome (LS) recommend molecular screening of colorectal cancers (CRCs) to identify patients for germline mismatch repair (MMR) gene testing. As our understanding of the LS phenotype and diagnostic technologies have advanced, there is a need to review these guidelines and new screening opportunities. We discuss the barriers to implementation of current guidelines, as well as guideline limitations, and highlight new technologies and knowledge that may address these. We also discuss alternative screening strategies to increase the rate of LS diagnoses. In particular, the focus of current guidance on CRCs means that approximately half of Lynch-spectrum tumours occurring in unknown male LS carriers, and only one-third in female LS carriers, will trigger testing for LS. There is increasing pressure to expand guidelines to include molecular screening of endometrial cancers, the most frequent cancer in female LS carriers. Furthermore, we collate the evidence to support MMR deficiency testing of other Lynch-spectrum tumours to screen for LS. However, a reliance on tumour tissue limits preoperative testing and, therefore, diagnosis prior to malignancy. The recent successes of functional assays to detect microsatellite instability or MMR deficiency in non-neoplastic tissues suggest that future diagnostic pipelines could become independent of tumour tissue.

## 1. Introduction

Lynch syndrome (LS) is the genetic predisposition to cancer in a variety of organs, in particular those of the gastrointestinal and genitourinary systems, caused by a germline pathogenic variant affecting one of four mismatch repair (MMR) genes: *MLH1*, *MSH2*, *MSH6*, or *PMS2* [1]. The most prevalent cancers within the Lynch spectrum are colorectal cancer (CRC) and endometrial cancer (EC), with cumulative incidences up to 57.1% and 48.9% by age 75 years, respectively, based on data from the Prospective Lynch Syndrome Database (PLSD) [2]. Known LS gene carriers can benefit from personalised cancer treatment, cancer surveillance, and cancer prophylaxis, including colorectal and gynaecological surgery and daily aspirin intake [1,3,4,5,6]. Hence, their identification is critical to optimise their clinical management. Since the discovery of its genetic cause in the 1990s, the strategies to identify LS have evolved with the advance of our knowledge of its phenotype and the diagnostic technologies available. However, LS is vastly underdiagnosed, with estimates that it is as common as one in 279 of the general population (*MLH1* = one in 1946, *MSH2* = one in 2841, *MSH6* = one in 758, and *PMS2* = one in 714) [7], and accounts for approximately 3% of CRCs and ECs [8,9]. Nearly three decades since its genetic definition, which have seen the completion of the Human Genome Project and the development of 2nd- and 3rd-generation sequencing strategies, a reappraisal of current LS screening strategies, and consideration of how these may evolve, is timely. Furthermore, the emergence of immunotherapy based on PD-L1 blockade has brought new impetus to the field, with pembrolizumab being the first site-agnostic agent to be licenced for cancer treatment. This has made identification of MMR-deficient tumours, whether sporadic or hereditary [3], a growing need in patient care. This will, in future, drive diagnostic development and with it, more frequent identification of LS probands.

### 1.1. Defining LS: A History

The first clinical description of LS was Alfred Scott Warthin’s “Family G” in 1913. Thirty three of its 70 members had been diagnosed with colonic, endometrial, or gastric cancer, suggesting an autosomal dominant inheritance of increased cancer risk [10]. Through the 1960s, 1970s and 1980s, Henry T. Lynch published a series of cancer families, including a follow-up description of Family G [11], and gave LS its first widely accepted name: Hereditary Non-Polyposis Colorectal Cancer (HNPCC). HNPCC was defined to identify families for linkage analysis, and to highlight the lack of polyposis in the colorectum in contrast with the well-described CRC syndrome Familial Adenomatous Polyposis (FAP). It was further subdivided into Lynch syndromes I and II depending on the tumour spectrum in the family [12].

In 1993, the underlying genetic cause of LS was discovered. Analysis of polymorphic microsatellites (short tandem repeat sequences) to detect loss of heterozygosity revealed that approximately 15% of CRCs have an exceptionally high frequency of microsatellite insertion and deletion variants (indels), a phenotype designated high microsatellite instability (MSI-H) or replication error positive. These MSI-H CRCs were diploid (unlike the majority of CRCs that show chromosomal instability), were associated with HNPCC, had a better prognosis, and tended to be proximally located (right sided) with poor cellular differentiation and increased immune cell infiltration [13,14,15,16]. Concurrently, in vivo experiments in yeast showed that loss-of-function mutations in MMR genes *MLH1*, *MSH2* and *PMS1* caused an MSI-H phenotype [17]. The causative link between MMR gene defects and LS was then established using the yeast *MSH2* gene to map human *MSH2* to chr2 p16-p21 and identify a pathogenic variant segregating with MSI-H HNPCC [18,19]. Pathogenic variants were subsequently found in the other MMR genes throughout the 1990s, including *MLH1* [20], *PMS2* [21], and *MSH6* [22]. In 2009, it was shown that 3′ deletions in *EPCAM* also cause LS through methylation of the deletion locus and silencing of the neighbouring MSH2 gene [23]. Sporadic MSI-H CRCs were shown to be associated with promoter methylation (and therefore silencing) of *MLH1* [24,25]. The term HNPCC has been replaced by “Lynch” syndrome to recognise the risk for a broad spectrum of tumours beyond CRC, and to unify Lynch syndromes I and II by their shared genetic aetiology [26].

### 1.2. Current Clinical Guidance for LS Screening

In 2017, the UK National Institute of Health and Care Excellence (NICE) published their Diagnostic Guidance 27, stating that all CRC patients, irrespective of age or other clinical features, should be screened for LS [27]. The multistep screening pipeline begins with molecular analysis of the CRC. MMR deficiency testing of the tumour, by MSI analysis or immunohistochemistry (IHC) to show loss of MMR protein expression, is used to identify potential LS-associated CRCs. The utility of MMR deficiency testing is based on the repeated observation that nearly all LS CRCs are MMR deficient [28,29,30,31], following somatic loss of function of the second allele of the germline-affected MMR gene according to Knudson’s two-hit hypothesis [32]. Subsequently, MMR-deficient CRCs are tested for *BRAF* c.1799T>A (p.V600E) variants and *MLH1* promoter methylation to improve screening specificity, as both are associated with sporadic tumours (*p* < 0.001) [33,34,35,36]. Patients with MMR-deficient CRCs lacking *BRAF* c.1799T>A (p.V600E) variants and *MLH1* promoter methylation are selected for germline MMR gene testing [27]. Similar screening guidelines have been published by the American Society for Clinical Pathology [37], and the European Society for Medical Oncology [38].

These guidelines are based on decades of evidence that show molecular tumour analysis is a superior screening strategy to select CRC patients for germline MMR gene testing compared to screening by familial or clinical criteria [1]. For example, pooled data from four large cohorts of unselected CRC patients (*n* = 3671), consulted between 1994 and 2010, found that the Bethesda Guidelines, which screen by familial and clinical criteria followed by molecular tumour analysis [39,40], had 87.8% sensitivity and 97.5% specificity for LS gene carrier detection, whereas screening by universal molecular tumour analysis had 100% sensitivity and 93.0% specificity [8]. Cost-effectiveness analyses, which balance the cost of patient screening and cascade testing of family members against the benefits of cancer surveillance and prophylaxis, further support screening strategies based on molecular analysis of CRCs [41,42,43]. A study comparison has shown general agreement of this cost-effectiveness between different countries [44]. An assumption made by these cost-effectiveness analyses is that the identification of LS probands and asymptomatic relatives who carry the LS variant will reduce cancer burden and costs due to prevention, surveillance and early detection [43]. Therefore, it is of critical importance to the clinical utility of LS screening that healthcare infrastructure has the capacity to offer these interventions to all identified LS gene carriers.

The implementation of LS screening in clinical practice has had mixed efficacy. A systematic review in 2017 identified five studies assessing the clinical performance of LS screening of CRC patients. Three of the five studies used a universal molecular screening approach, and two preselected patients based on clinical and familial criteria prior to molecular analyses of the tumour. The frequency of LS diagnoses ranged from 0.0% (0/31) to 5.3% (3/57) [45]. The largest study employing universal screening had an LS detection rate of 2.2% (17/784) [46], which suggests that approximately 73% of LS gene carriers were identified assuming 3% of the cohort were carriers. This shows current diagnostic guidance can be effective in a clinical setting. However, in the US, between 2010 and 2012, only 28.2% (43,143/152,993) and 43.1% (7422/17,218) of CRC patients aged below 70 and 50 years, respectively, were tested for tumour MMR deficiency [47], despite concurrent estimates that only 1.2% of LS gene carriers were known to clinical services [48]. Similarly, estimates from the UK suggest that, in 2016, routine screening for LS in CRC patients aged below 50 years was not performed in 29.5% (46/156) of UK hospitals [49] despite guidance promoting this from the UK Royal College of Pathologists [50]. Given these observations, here, we review the key barriers to implementation, as well as the limitations, of current LS screening guidelines. Following this we discuss the advances in our technology and knowledge that may further improve LS identification by addressing these barriers and limitations, or providing new screening opportunities.

## 2. Barriers to Implementing LS Screening Guidance

A US survey of 509 clinicians belonging to the American College of Gastroenterology found that the most common reasons given for a lack of MMR deficiency testing of CRCs to screen for LS were: prohibitive cost (33.3%), unfamiliarity interpreting results (29.2%), unavailable genetic counselling (24.9%), and unavailable germline genetic testing (20.0%) [51]. Similarly, cost, practical limitations, resources, and genetic counsellor availability were the key limitations identified in UK hospitals [49] and among Canadian genetic counsellors and pathologists [52]. These surveys show that the follow up of patients with a CRC suggestive of LS has barriers that are equally important to address as the barriers to universal molecular analysis of CRCs. This is also evident in clinical studies assessing the efficacy of LS screening in practice. For example, in a study of 1612 CRCs tested for MMR deficiency during the period 2004–2013, only 29.9% (82/274) of patients with a MMR-deficient tumour were subsequently consulted at a familial cancer clinic, leading to a low yield of LS diagnoses at 0.6% (10/1612) [53]. Uptake of LS screening within different demographics, particularly the clinically underserved, may face additional barriers, including access to clinics, cultural beliefs around healthcare, and language barriers [54]. Indeed, an analysis of CRC patient outcomes from 2012 to 2016 in four US centres showed that, whilst there was no difference in tumour MMR deficiency testing uptake or results, African American and Hispanic patients were significantly less likely to be referred for genetic counselling and testing [55]. Specialist hospitals setup to address such specific needs have been shown to provide high-quality LS screening to >90% of patients irrespective of their background [54]. However, a concerted effort to overcome both general and demographic-specific barriers will be needed for fair and widespread LS screening to be achieved. The suggestion of dedicated LS screening programs has had strong support from healthcare professionals [56]. Careful program and policy design, an interdisciplinary approach, and sustained funding have been highlighted as the key requirements of a successful LS screening program [52].

Stakeholder education will be an important consideration when deploying LS screening programs. A 2016 survey of UK National Health Service General Practitioners found that 29.2% (294/1007) were not aware of LS (including by its alternative names such as HNPCC), and only 46.7% of those who had heard of LS were aware of the reduction in CRC risk associated with daily aspirin intake [57]. Another survey found that 41% (82/201) of US medical students did not know of LS. Of the students who had heard of LS, only 46% knew its genetic aetiology, only 23% knew of screening criteria to identify LS, and only 32% and 17% knew of recommendations for CRC and EC surveillance, respectively [58]. A lack of knowledge of the LS phenotype and clinical management was also observed in a 2014 survey of Australian healthcare providers from a variety of disciplines likely to encounter an LS patient: 7.7% (11/144) were unfamiliar with hereditary cancer syndromes in general, and 13.4% thought guidelines for LS screening were unavailable [59]. In this same study, the most frequently (55.6%; 79/142) identified barrier to referring suspected LS gene carriers for further testing was a lack of interest from the patient [59]. This suggests that even with an optimised and informed healthcare service, the uptake of LS screening may still be limited. Therefore, patients are also a key target of stakeholder education. In a randomised controlled trial of universal MMR deficiency testing of CRCs to screen for LS, compared to physician- or self-referral, a survey of the 145 participants’ perspectives showed that more than 90% agreed that universal LS screening should be offered to CRC patients, and that they understood the reason for screening. Furthermore, when given a list of potential benefits of LS screening (for example, better understanding of hereditary CRC risk), 50.3% endorsed all eight benefits and 84.8% endorsed at least six benefits. Very low levels of anxiety due to screening were observed [60]. This is in contrast to the low patient interest observed by some healthcare practitioners [59]. The discrepancies of these studies may, in part, be explained by the composition of the patient population and the information provided to them: Clinical trial participants will have been provided high-quality information curated by healthcare professionals with an interest in LS screening, and likely represent a more engaged patient population. This suggests that patient education is likely to have a major impact on perceptions and uptake of screening.

Barriers of finance, logistics, and education may be addressed by government investment in genetics healthcare. Not only do such investments build clinical infrastructure and sequencing capacity, from which dedicated LS screening pipelines will benefit, they also popularise genetics. The 100,000 Genomes Project (100 kGP) has had a significant impact on genetics services in the UK. Initial challenges faced by the 100 kGP included pipelines for DNA extraction, establishing quick turnaround times from DNA sample receipt to analysis, and data storage and access. Whilst the utility of whole-genome sequencing to direct patient care is not agreed by all clinicians, the 100 kGP has demonstrated the feasibility of turnaround times <18 days, and has established data management systems that include secure links to patients’ clinical records and several national datasets [61]. Large-scale initiatives also provide an opportunity to explore associated ethical, legal, and social issues, such as the impact on different peoples (particularly the clinically underserved), accountability within the clinical pathway, and the patients’ privacy, data security, and role as stakeholders [62], all of which will be informative for LS screening programs.

## 3. Limitations of LS Screening Guidance

Beyond the financial and logistical barriers to implementation, it is also important that stakeholders are aware of the limitations of these guidelines to inform current practice as well as future screening strategies.

### 3.1. Not All Colorectal Cancers Are Made Equal

Current LS screening guidelines use cost-effective and highly sensitive molecular tests of CRCs to select patients for germline MMR gene analysis. However, not all LS CRCs fit the archetype that these guidelines are based on. Multiple studies using IHC, MSI analysis, and/or sequencing have shown that up to 6.9% (54/58; 95% CI: 83.3–98.1%) of CRCs that develop in LS gene carriers are MMR proficient [28,29,30,31]. Given the variety of assays used and the consistency of these observations, this likely represents the development of sporadic MMR-proficient tumours rather than test insensitivity. Therefore, current CRC-based screening guidelines cannot be 100% sensitive for LS detection.

Interestingly, there is growing evidence that colorectal tumorigenesis in LS is heterogeneous, depending on which MMR gene is affected in the germline. For instance, adenoma rates in carriers of pathogenic *MLH1* variant carriers have been shown to be significantly lower than in pathogenic *MSH2* variant carriers, despite equal CRC incidence [63]. Further, mutations in *CTNNB1*, previously associated with a non-polypous pathway of tumour progression [64], are prevalent in MLH1-deficient but not MSH2- or MSH6-deficient LS CRCs [63]. MMR deficiency may also be a less prevalent driver of tumorigenesis in carriers of pathogenic *MSH6* or *PMS2* variants as they have a much lower CRC incidence than carriers of pathogenic *MLH1* or *MSH2* variants [2]. Due to this heterogeneity, it is probable that the frequency of MMR deficiency in LS CRCs, and hence the sensitivity of current screening guidance, varies by the affected gene. Indeed, in line with a lower CRC incidence, it has been shown that oncogenic driver mutations in the majority of CRCs from pathogenic *PMS2* variant carriers are not related to mutational signatures of MMR deficiency [65], which is in contrast to driver mutations in the majority of LS colorectal tumours associated with other MMR genes [66,67]. Furthermore, only 16.7–36.4% of colorectal adenomas from pathogenic *MSH6* variant carriers were shown to be MMR deficient, much lower than the 63–92% of adenomas from *MLH1* or *MSH2* variant carriers in the same studies [68,69]. In agreement, a pan-cancer analysis of MSI found that 78.4% (29/37) of LS-associated microsatellite stable tumours were from carriers of either *MSH6* or *PMS2* pathogenic variants [70]. Additional research into the impact of these heterogeneous pathways on LS identification strategies would be informative.

Patients with MMR-deficient CRCs can also be excluded from LS screening pipelines following positive *BRAF* c.1799T>A (p.V600E) or *MLH1* promoter methylation test results. A recent analysis found that *BRAF* c.1799T>A (p.V600E) is present in 1.6% (15/969; 95% CI: 0.9–2.5%) of LS MSI-H CRCs, and, where age data were available, 85.7% (6/7; 95% CI: 42.1–99.6%) of these were from patients aged < 50years. In contrast, 92.3% (313/339; 95% CI: 89.0–94.9%) of *BRAF* c.1799T>A (p.V600E)-positive sporadic MSI-H CRCs were from patients aged > 60 years, suggesting that *BRAF* c.1799T>A (p.V600E) result interpretation could be refined based on patient age [71]. Presence of *MLH1* promoter methylation can also exclude LS patients from screening pipelines, either as the second hit in *MLH1*, which occurs in 15.8% (3/19; 95% CI: 3.4–39.6%) of CRCs arising in pathogenic *MLH1* variant carriers [72], or as a germline *MLH1* epimutation, which accounts for 2–3% of *MLH1*-asscociated LS [73,74,75]. Therefore, whilst molecular screening of CRCs is a highly sensitive strategy for LS detection, clinical features of the patient, such as young age and family history, may warrant germline MMR gene testing irrespective of tumour test results.

### 3.2. Ambiguous Genetic Diagnoses

A definitive diagnosis of LS requires detection of a germline pathogenic MMR variant. A definitive diagnosis can be complicated by variants of unknown significance (VUS), pseudogenes of *PMS2*, and a failure of current methodology to detect the causative variant. A 2016 summary of MMR variants in the InSiGHT database found that VUSs accounted for 31% of all variants recorded [75]. Analysis of tumour MMR or MSI status can help reinterpret MMR VUS as likely pathogenic or likely benign [76,77]. However, additional assessments are needed to confirm variant classifications. In silico analyses, which predict variant pathology by integrating clinical data and predicted changes to RNA and protein sequence, can be used to prioritise VUSs for functional assessment, as demonstrated by in silico re-classification of 53.7% (29/54) of MMR VUS as probably damaging in one single centre study [78]. Such in silico analyses can then be followed up by assays of MMR function in the presence of the VUS. In vivo functional assays are possible due to the highly conserved structure and function of the MMR system, allowing model organisms, such as yeast or mice, to be used. In one example, oligonucleotide-directed mutagenesis was used to introduce *MSH2* VUSs into mouse embryonic stem cells. Subsequent selection of MMR-deficient clones by 6-thioguanine treatment (a guanine analogue that, when incorporated into genomic DNA, initiates cell cycle arrest and apoptosis through MMR), followed by sequencing of resistant clones to show selection of the VUS, determined that 32.2% (19/59) of VUS tested were pathogenic. Furthermore, the assay was able to differentiate between variants that fully disrupted MMR and those that only attenuated it [79]. Such in vivo assays require specialist setups that are not generally available to diagnostic laboratories. An alternative, the complete in vitro MMR activity (CIMRA) assay, uses in vitro transcription and translation to produce MMR protein containing the VUS of interest. This is added to a nuclear extract lacking the corresponding MMR protein, along with a DNA substrate containing a T-G mismatch. Repaired substrate is specifically cleaved by *HinD*III restriction enzyme, and is quantified relative to a wild-type control. By comparing this to the repair activity of known pathogenic or benign variants, the odds of pathogenicity for a VUS can be determined and combined with other probabilistic predictors of pathogenicity [80,81]. Using such assays, large-scale and coordinated efforts, with dedicated annotation of variant databases, will be needed to process the large number of MMR VUS remaining.

Analysis of *PMS2*, located on chromosome 7 and containing 15 exons, is complicated by the presence of 13 pseudogenes situated on the same chromosome [82]. The largest of these, *PMS2CL*, an approximate 100 kb inverted duplication, shares ~98% identity to *PMS2* exons 9 and 11–15. The remaining 12 pseudogenes each have >90% identity to regions in the 5′ end of *PMS2*. These mean that 6.7% of the gene is a ‘dead-zone’ of sequencing due to ambiguous capture and read-alignment [83]. Multiple techniques have been developed to address this. Long-range PCR, with primers targeting unique sequences, can be used to generate gene-specific products for subsequent exon amplification and Sanger sequencing. In one analysis, this method identified 10 novel and 17 previously detected variants from 30 colorectal and 11 endometrial cancer patients, including five novel pathogenic variants [82]. Alternatively, cDNA sequencing from short term lymphocyte cultures (puromycin-treated to prevent nonsense-mediated decay of mRNA) can differentiate between *PMS2* and its pseudogenes, detect splice variants, and detect *PMS2/PMS2CL* ‘hybrid’ alleles, which may account for ~10% of all *PMS2* alleles in the European population [84]. However, the scalability of both methods is limited by non-automatable protocols.

### 3.3. Lynch-Like Syndrome

Approximately one-half to two-thirds of MMR-deficient and *MLH1* promoter methylation-negative CRC patients do not have a germline pathogenic MMR variant [85,86]. Families of patients with these Lynch-like CRCs were found to have a significantly higher risk of CRC than the families of sporadic CRC patients, but a lower risk than LS families, and hence this milder cancer predisposition was termed Lynch-like syndrome (LLS) [87]. An early study of LLS cases found that 52.0% (13/25) were explained by double somatic pathogenic variants in *MLH1* or *MSH2* [88], and later studies analysing all four MMR genes have shown that approximately 73.0% (92/126; 95% CI: 64.4–80.5%) can be explained by double somatic pathogenic variants in any MMR gene [86]. CRCs with double somatic pathogenic MMR variants are histologically indistinguishable from MMR-deficient LS CRCs [89], but can be identified by paired tumour and germline MMR gene sequencing [76]. Whilst lacking *MLH1* promoter methylation, these CRCs may also have a higher frequency of CpG island methylator phenotype compared to LS CRCs (13/14 versus 9/18; *p* = 0.019) [85]. However, the familial association of LLS and the cause of MMR deficiency in >15% of LLS CRCs are currently unexplained [85,86,87].

As discussed by Carethers [90], the intermediate CRC risk of LLS likely represents a heterogeneous population of unidentified LS cases, sporadic cases, and other familial CRC predisposition syndromes that have acquired MMR deficiency independent of the causative germline variant. The intermediate risk for other Lynch-spectrum tumours in the families of LLS patients [91] further supports that some are unidentified LS gene carriers. A failure to detect germline MMR gene variants may be due to limitations of technology and our understanding of MMR genetics and regulation. A few examples follow: large structural changes affecting MMR genes can be missed by conventional (Sanger or short-read next-generation) sequencing, such as the Finnish *MLH1* founder variant, a 3.5 kb deletion of exon 16 and flanking intronic sequence [85,92], or an inversion of *MSH2* exons 2–6 discovered in Australian LS patients [93]. Epimutations can also be missed by conventional sequencing methods, such as the methylation and silencing of *MSH2* due to deletions in the 3′ end of *EPCAM* [23], which accounts for approximately 2.8% and 1.1% of LS families in The Netherlands and Germany, respectively [94]. Epimutations also cause 2–3% of *MLH1*-associated LS [73,75]. Long-range gene expression regulation may account for some missed LS cases. For instance, a 1.8 kb enhancer of *MLH1* expression, positioned 35 kb upstream of the transcription start site, has been shown to contain a single nucleotide variant (rs143969848) capable of disrupting enhancer function that was found in 5.4% (4/74) of patients suspected to carry (based on tumour analysis), but lacking, a germline pathogenic *MLH1* variant [95]. Somatic mosaicism is another rare cause of LS, diagnosis of which requires non-standard techniques (for example high depth sequencing) to detect low variant allele frequencies in constitutional tissues [96,97,98]. Somatic mosaicism combined with other rare features, such as epimutation, may further complicate diagnosis [74]. Carethers [90] also highlighted that mechanisms outside of the core four MMR genes could affect MMR function, and, therefore, pathogenic variants in other genes could be an unknown cause of LS.

Recently, Dámaso et al. used sequencing of a custom 26 CRC gene panel and genome-wide methylome analysis of germline DNA to explore the frequency of these diagnoses among 115 LLS patients. They found that eight had germline MMR pathogenic variants that had previously been missed or misclassified, and one had a constitutional *MLH1* epimutation, confirming a diagnosis of LS in nine (7.8%). Twelve (10.4%) had a germline pathogenic variant in another CRC-associated non-MMR gene, indicating that the patient had an alternative CRC predisposition syndrome with somatic MMR deficiency in the tumour [99]. Double somatic MMR variants have also been found in the CRCs of patients with polymerase proofreading-associated polyposis (PPAP) [100,101], providing further evidence that MMR deficiency can be found in hereditary CRCs that are not caused by germline MMR gene variants. PPAP is caused by germline pathogenic variants in the exonuclease domains of *POLD1* or *POLE* genes, and affected families have an increased risk for a number of tumours also prevalent in LS, in particular CRC and EC. The polyposis phenotype is varied, ranging from few or no polyps equivalent to LS, to densities seen in FAP and other polyposis syndromes. The CRCs from these patients present with hypermutation due to a failure of polymerase proofreading [102,103], which can cause subsequent mutation of MMR genes, MMR deficiency and increased MSI, hallmarks of a Lynch-like CRC [100,101]. Therefore, PPAP, and other CRC-predisposition syndromes, will account for a currently unknown fraction of LLS cases.

## 4. The Next Generation of LS Screening

Since the 1990s, LS screening strategies have developed with new knowledge and with new technologies.

### 4.1. Advances in Tumour MMR Deficiency Testing

A key advancement in tumour MSI testing was the move from marker panels of mixed microsatellite motifs, such as the National Cancer Institute reference panel that included two mononucleotide repeats (monoNRs) and three dinucleotide repeats (diNRs) [104], to panels exclusively containing monoNRs. There are several reasons for this. Primarily, MSH6-deficient tumours were frequently misclassified as microsatellite stable or microsatellite instability-low, which are both biomarkers of MMR proficiency [105,106,107], as the MSH2-MSH6 heterodimer (MutSα) of the MMR system only maintains monoNR stability and not the stability of microsatellites with longer repeat motifs [108]. Panels of monoNRs have been shown to correctly classify 97.7–100% of MSH6-deficient CRCs, ECs, and urothelial cancers as MSI-H [109]. Furthermore, amplification of monoNRs has been shown to have a higher fidelity than amplification of diNRs [110], and monoNR panels have been used to re-classify MSI-L tumours as MSS due to removal of ambiguous diNR results [111]. A multiplexed, fluorescently-labelled PCR of five monoNRs, with amplicon fragment length analysis (FLA) by capillary electrophoresis, achieved near 100% accuracy for MMR deficiency detection [112], and was developed into the widely used MSI Analysis System (Promega). Similarly, it is now considered best practice to stain for all four MMR proteins when using IHC to assess MMR status [113], partly due to the degradation of PMS2 that fails to complex with non-functional but antigenic MLH1 protein [114,115]. 

More recent technological advances may address some of the barriers and limitations of tumour MMR deficiency testing. For example, the Idylla MSI test (Biocartis), a PCR-based assay developed for ease of use and a rapid turnaround time, has recently been shown to have 99.7% concordance with the MSI Analysis System (Promega) in 330 CRCs [116]. Another recent study used large, international cohorts to show that neural network analysis of haematoxylin and eosin stained tumour tissue sections can detect MMR deficiency in CRCs with a ROC AUC of 0.95 [117]. One of the biggest advances in the last decade is the advent and decreasing cost of massively parallel sequencing technologies, which has provided several new tools to identify LS gene carriers. “Next-generation” sequencing (NGS) has several advantages relative to conventional methods, and can be applied to either tumour analysis or germline MMR gene testing. For example, NGS gene panels have been shown to detect base substitutions, indels, copy number variations, and gene fusions across 287 genes with 95–99% sensitivity and >99% specificity, and to identify clinically actionable variants in 76% of cancers, approximately 3-fold more than established, non-NGS-based biomarker tests [118]. This allows multiplexed and automated analysis of many genetic biomarkers in (potentially) hundreds of samples simultaneously, whilst using simple laboratory protocols for sequencing library preparation.

### 4.2. Tumour Sequencing to Screen for LS

A wide variety of cancer gene panels are now available, the majority of which include the MMR genes as well as *BRAF*. Where MMR VUS are detected, functional data from MSI analysis or IHC can resolve diagnostic uncertainty [29,76,77]. Software for MSI analysis of microsatellites captured by gene panel (or more extensive) sequence data are available [119,120,121,122,123]. In the original publications, and in comparative testing [122,123], each software had sensitivities and specificities >95% for MMR deficiency, making them comparable to MSI FLA and IHC. Therefore, tumour sequencing can provide a more direct and multifaceted tool for LS screening than conventional molecular tumour assays. Furthermore, the identification of double somatic MMR mutations by tumour sequencing avoids managing approximately half of Lynch-like tumour patients as LLS [76,88]. However, tumour gene panel (or more extensive) sequencing suffers significant barriers to widespread deployment. These include cost [124], the large number of VUS that can be found [125], and the storage, processing, and interpretation of big data, which require specialist personnel [126]. Alternatively, MSI assays that enrich and sequence specific microsatellites can be used, keeping costs equivalent to or less than MSI FLA or IHC, whilst leveraging the scalability and automatability of NGS and multiplexed analysis of *BRAF* c.1799T > A. Such assays can reduce LS screening to one, low cost, high throughput tumour test prior to germline MMR gene testing of select cases, and could facilitate widespread deployment of current guidance [127,128,129].

A number of recent studies have shown that LS screening pipelines that use tumour sequencing have a higher sensitivity than pipelines that use conventional molecular screening methods [30,76]. For example, Hampel et al. selected patients with pathogenic variants in at least one allele of an MMR gene in their tumour for germline MMR gene testing from 419 unselected CRC patients, identifying twelve (2.9%) LS gene carriers (2 *MLH1*, 5 *MSH2*, 1 *MSH6*, 4 *PMS2*). These were supplemented with an additional 46 known LS gene carriers to compare this tumour sequencing-based approach to conventional molecular screening. In total, 93.1% (54/58) of LS CRCs were MSI-H by PCR FLA, and 91.4% (53/58) were MMR deficient by IHC. mSINGS [120] analysis of the tumour sequence data was 99.8% concordant with MSI FLA. One of the LS gene carriers had methylation of the *MLH1* promoter in addition to MSI-H in their tumour, and therefore would have been excluded from LS screening by conventional molecular analysis pipelines. This patient was not excluded by the tumour sequencing pipeline as *MLH1* promoter methylation was not tested, and the tumour was *BRAF* c.1799T>A (p.V600E) negative [30]. Therefore, adoption of tumour sequencing in LS screening pipelines may help overcome the previously highlighted barriers and limitations to MMR deficiency testing, specifically testing capacity, results interpretation, and reduced sensitivity due to MMR-proficient CRCs.

### 4.3. Germline Sequencing to Identify LS

In their 2014 cost-effectiveness analysis of different LS screening strategies, Snowsill et al. found that direct germline MMR gene testing of all CRC patients aged <60years was a cost-effective intervention [43]. A further consideration is that approximately 9.9% of all CRCs are associated with known germline pathogenic variants, and only one-third of these are in MMR genes [29]. Therefore, immediate germline gene panel sequencing of CRC patients would facilitate detection of CRC predisposition syndromes beyond LS, and may be a more cost-effective intervention. For example, Gu et al. created a model to compare the cost-effectiveness of germline gene panel sequencing to current LS screening guidelines in CRC patients with >5% probability of hereditary cancer based on a predictive, clinical algorithm, and US epidemiology. Their model predicted that germline gene panel sequencing had a cost-effectiveness of $1543 per quality adjusted life year (QALY) gained, whilst LS screening alone was less cost-effective at $1882 per QALY gained [130]. Whilst widespread adoption of universal germline gene panel (or MMR gene) sequencing is currently unlikely due to its limitations (discussed above), it may impact LS screening strategies in the near future as the demand for diagnostic sequencing increases, and sequencing technology becomes cheaper and more accessible.

New sequencing technologies may also address some of the limitations of germline MMR gene testing, such as the detection of the undetected LS cases that likely make up a substantial portion of LLS [90]. A development that holds much promise is long-read sequencing, which generates reads spanning kilobases of a single molecule to overcome some of the limitations of traditional short-read sequencing [131,132]. For example, short reads are generally considered to have a poorer sensitivity for detecting structural variants than long reads [133]. Recently, long-read sequencing was used to clarify suspected structural variants in the germlines of hereditary cancer patients [134], and there are known examples of large deletions or rearrangements causing LS, such as the Finnish *MLH1* founder variant [85,92] and Australian *MHS2* inversion [93] described earlier. In addition, novel base-callers being developed for long-read sequencing data may be able to detect base modifications from subtle effects on sequencing dynamics [132], which could facilitate detection of germline epigenetic modifications of MMR genes, such as those estimated to account for 2–3% of *MLH1*-associated LS [73,74,75]. Long-read sequencing may also resolve ambiguous variant calls from genes that have high homology pseudogenes such as *PMS2* [82,131], variants in which may be the most prevalent cause of LS [7]. Therefore, long-read sequencing may have a significant impact on LS diagnostics in the near future through the detection of currently unknown MMR gene structural and epigenetic variants, and unambiguous analysis of *PMS2*, among other applications.

## 5. Should LS Screening Include Additional Lynch-Spectrum Tumours?

Large-scale longitudinal studies established within the last decade have allowed us to more accurately determine the cancer risks of LS gene carriers, and better define the Lynch spectrum of tumours. The other tumours, in addition to CRC and EC, generally agreed to be within the Lynch spectrum are those of the upper gastrointestinal tract (stomach, biliary tract, pancreas, and small bowel), urothelial tract (bladder, ureter, and kidney), ovaries, central nervous system (CNS), and sebaceous glands [1]. There has been debate as to whether breast and prostate cancer fall within the Lynch spectrum [1]. Recent prospective evidence suggests that carriers of pathogenic *MSH2* variants in particular have an increased risk of prostate cancer (PrC) relative to the general population, and a slight increase in breast cancer (BC) risk has been observed irrespective of which MMR gene is affected [2]. For this review, we do not include PrC and BC within the Lynch spectrum of tumours, but discuss these separately.

Based on the cumulative cancer incidence by 75 years from the PLSD [2], it can be surmised that, depending on sex and which MMR gene is affected in the germline, CRCs account for only 24.1–62.5% of Lynch-spectrum cancer diagnoses. Therefore, up to three-quarters of LS gene carriers that present with a Lynch-spectrum cancer are not being screened for LS following current guidance. Furthermore, in LS gene carriers, CRC is often not the sentinel cancer, particularly among females, where the first cancer is most often EC [135]. For example, in a recent study of LS patients with gastric cancer (GC), 39.0% (16/41) had the GC as their first malignancy, and in 9.8% (4/41) GC was their only Lynch-spectrum tumour [136]. In another study, in 32.3% (10/31) of LS patients with small bowel cancer (SBC), the SBC was their first cancer; in a further 12.9% (4/31), the SBC was their first cancer with a synchronous CRC; and in 19.4% (6/31), the SBC was the only cancer diagnosis [137]. Similarly, in one cohort of bladder urothelial cancer (BUC) patients with LS, the BUC was the first cancer in 38.1% (8/21) of patients [138]. A failure to detect LS in patients whose first cancer is not CRC means clinical management cannot be provided to reduce their risk of additional, preventable cancers.

### 5.1. Molecular Analysis of Endometrial Cancers Is a Clinically Useful Screen for LS

Female LS gene carriers have an equivalent or higher risk for EC than CRC [2], and, in approximately half, EC is their first cancer [135]. The Manchester International Consensus Group is leading an effort to provide clinical guidance for both the screening of ECs to identify LS and the management of ECs and EC-risk in known LS gene carriers [139]. Clinical and familial criteria are poor predictors of LS in EC patients, with a recent meta-analysis finding that 43% of LS diagnoses in EC patients would be missed following this strategy [140]. Alternatively, multiple studies have shown that molecular screening of ECs has a superior sensitivity for LS detection [141,142,143,144,145]. A recent meta-analysis, encompassing 12,633 patients, has shown that 25–28% of ECs are MMR deficient and that approximately 3% of all ECs are associated with LS [9], frequencies that are equal to or even higher than CRC. IHC and MSI analysis are both suitable tests of MMR status in ECs, with high sensitivity and specificity, and approximately 95% concordance between them according to several independent studies [145,146,147,148]. However, some studies report a lower sensitivity of MSI testing [143,149]. The MSI signal in ECs may be weaker than in CRCs, with a median 126 unstable microsatellites detected in ECs compared to 290 in CRCs [150], and observations of a mean 3 nucleotide deletion shift in microsatellites of MSI-H ECs versus 6 nucleotides in CRCs [151]. Furthermore, among LS ECs, loss of MSH6 may be the most common cause of MMR deficiency [141,143,145], whilst loss of MLH1 or MSH2 predominates in LS CRCs [8,125]. This means that only monoNRs should be used for MSI analysis of ECs. Therefore, a weaker signal, use of outdated microsatellite marker panels, and differences in interpretation method may account for the reported lower sensitivity of MSI analysis in some studies.

As for CRCs, the specificity of molecular LS screening in EC patients can be increased by *MLH1* promoter methylation testing, and has been shown to identify approximately 94% of sporadic MMR-deficient ECs [152]. However, *BRAF* c.1799T>A (p.V600E) mutations are exceptionally rare in EC (0.1%; 4/2675; 95% CI: 0.0–0.4%) and testing for this mutation has no value for LS screening in ECs [152]. Another similarity to LS screening in CRCs is the presence of Lynch-like ECs that are caused by double somatic MMR mutations, which, as for CRCs, account for approximately half (12/22) of tumours selected for germline MMR gene testing [145]. Furthermore, MMR deficiency followed by *MLH1* promoter methylation testing of ECs to select patients for germline MMR gene testing is a cost-effective intervention [153,154]. Despite this evidence, a survey of UK gynaecological oncologists found that only 36.6% (16/41) were familiar with LS management guidelines, and only 29.3% agreed with offering LS screening to all EC patients. The most frequent reasons for disagreeing with universal screening of EC patients were a lack of funds, perceived low yield, and possibility of patient distress [155]. Other barriers to screening EC patients include a lack of genetic counselling and the management of uninformative genetic test results [156]. Overall, the sensitivity, specificity, and cost of (as well as barriers against) molecular screening of ECs to identify LS mirror what has been observed in CRC, suggesting that current guidance for LS screening in CRCs is applicable to ECs.

### 5.2. The Frequency of LS Is Equivalent across Most Lynch-Spectrum Tumours

Whilst the adoption of LS screening in EC patients is progressing with a building evidence base, less attention has been given to the other Lynch-spectrum tumours. Here, we have explored whether it is justifiable to expand LS screening to include other Lynch-spectrum tumour types beyond CRC and EC using evidence from the published literature. To assess the potential yield of screening for each tumour type, the frequency of LS in unselected tumour patients was collated and totalled from peer-reviewed publications (Table 1) using the detection of a pathogenic germline. MMR gene variant as the diagnostic criterion. Overall, LS was detected in 2.4–5.0% of patients for the majority of Lynch-spectrum tumours, equivalent to or greater than the 3.0% (95% CI: 2.7–3.3%) and 2.7% (95% CI: 2.3–3.1%) observed in CRCs and ECs, respectively, and significantly greater than the 0.3% estimate of LS in the general population [7]. Sebaceous neoplasms (SNs) were exceptional, with 33.3% (95% CI: 27.6–39.4%) of patients having LS. This high yield suggests that direct germline MMR gene testing of any SN patient should be offered. Another exception was ovarian cancers (OCs), in which only 1.4% (95% CI: 1.1–1.9%) of patients were found to have LS. However, several of the studies included patients whose tumours were of a serous histology, which is not associated with LS [157]. One study focusing on endometrioid or clear cell OCs found LS in 4.4% (4/90; 95% CI: 1.2–11.0%) of patients, suggesting that histological selection of OCs could increase the LS yield [158]. These frequencies support offering universal LS screening to biliary tract cancer (BTC), endometrioid OC, SN, SBC, and urothelial cancer (UC) patients.

GCs and pancreatic cancers (PaCs) had 1.0% (3/308; 95% CI: 0.2–2.8%) and 1.1% (41/3608; 95% CI: 0.8–1.5%) LS diagnoses among unselected patients, respectively, which are lower than the other Lynch-spectrum tumours. However, the frequency in PaCs is enriched compared to the 0.3% frequency that would be expected of the general population [7], and so LS screening in PaCs could be considered. More data from GCs are needed to determine whether the enrichment observed here is significantly greater than the expected population frequency. The final Lynch-spectrum tumour type, those of the CNS, only had a 0.3% (3/923; 95% CI: 0.1–0.9%) frequency of LS. However, this frequency came from only one study with no subtyping of CNS tumours, and therefore LS screening of CNS tumour patients should not be excluded based on this observation alone. Beyond the classic Lynch-spectrum of tumours, an LS frequency of 0.3% (11/3011; 95% CI: 0.2–0.7%) was observed among BC patients, equivalent to the 0.3% frequency of the general population, suggesting that LS in BC patients is an incidental finding. However, 1.2% (68/5831; 95% CI: 0.9–1.5%) of PrC patients were found to have LS, a frequency that is equivalent to that observed in PaC and GC and significantly greater than the general population frequency. This enrichment suggests that PrC is of the Lynch spectrum and LS screening could be considered in PrC patients. As expected, the frequency of LS among unselected non-Lynch-spectrum tumours was equivalent to the 0.3% frequency estimated for the general population at 0.4% (32/9116; 95% CI: 0.2–0.5%).

### 5.3. MMR Deficiency Is a Sensitive Biomarker of LS in All Lynch-Spectrum Tumours

Several historic studies showed that MMR deficiency is prevalent in the Lynch-spectrum tumours of LS patients [28,137,186,187,188], although the sample numbers in these studies were relatively low. More recently, increased MSI has been shown to associate with LS across all cancer types in a large study of 15,045 independent patients [70]. Therefore, it would be informative to explore whether MMR deficiency testing of other tumour types could be used to screen for LS as it is for CRCs, and as is suggested for ECs. Here, the frequency of MMR deficiency in tumours detected in known LS gene carriers was collated from published studies to explore the probable sensitivity of LS screening by universal MMR deficiency testing of each Lynch-spectrum tumour (Table 2). MMR deficiency was detected in 96.4% (95% CI: 91.9–98.8%) and 80.6% (95% CI: 71.4–87.9%) of LS CRCs and ECs, respectively. LS BTCs, OCs, and SBCs were MMR deficient in 93.8–100.0% of tumours, equivalent to LS CRCs. The frequency of MMR deficiency in LS CNS tumours, GCs, OCs, PaCs, SNs, and UCs ranged from 73.9 to 89.6%, similar to the frequency in LS ECs. These frequencies suggest that universal MMR deficiency testing of all Lynch-spectrum tumours would have an equivalent sensitivity for LS detection as MMR deficiency testing of CRCs and ECs. Overall, the frequency of MMR deficiency in non-Lynch-spectrum tumours from LS gene carriers was 18.2%. Although inconclusive due to low numbers, it is possible that different non-Lynch-spectrum tumours have different frequencies, with 100.0% (2/2; 95% CI: 15.8–100.0%) of LS oesophageal cancers being MMR deficient compared to 0.0% (0/6; 95% CI: 0.0–45.9%) of LS lung cancers. Interestingly, 50.9% (43/84; 95% CI: 40.0–62.3%) of LS BCs and 65.7% (47/72; 95% CI: 53.1–76.1%) of LS PrCs were MMR deficient, frequencies that are between those observed in classic Lynch-spectrum tumours and non-Lynch-spectrum tumours, correlating with the marginal increased risk for these tumours in LS gene carriers [2].

### 5.4. The Accuracy of LS Screening by MMR Deficiency Testing Is Comparable across Most Lynch-Spectrum Tumours

Finally, we extracted the frequency of MMR deficiency in unselected tumours of different types from published studies (Table 3) to allow a more comprehensive comparison of the outcome of testing between each tumour type. The frequency of MMR deficiency in unselected Lynch-spectrum tumours, other than CRC and EC and excluding CNS tumours, ranged from 2.5% (224/8954; 95% CI: 2.2–2.8%) in PaCs to 42.2% (584/1385; 95% CI: 39.5–44.8%) in SNs, with several tumour types having frequencies similar to the 13.9% (1823/13093; 95% CI: 13.3–14.5%) and 26.6% (1735/6522; 95% CI: 25.5–27.7%) observed in CRCs and ECs. These frequencies are all significantly greater than the frequency in non-Lynch-spectrum tumours, which was 0.8% (55/7222; 95% CI: 0.6–1.0%). Only 1.0% (10/980; 95% CI: 0.5–1.9%) of CNS tumours were MMR deficient, which is not significantly greater than non-Lynch-spectrum tumours. However, as discussed below in Section 5.5, the true rate of MMR deficiency in CNS tumours may be higher. 1.7% (58/3319; 95% CI: 1.3–2.3%) of BCs and 1.0% (17/1674; 95% CI: 0.6–1.6%) of PrCs were MMR deficient. For BCs, this is marginally higher than in non-Lynch-spectrum tumours, whilst the slight increase in PrCs was not significantly different compared to non-Lynch-spectrum tumours.

We subsequently used these frequency estimates (Table 3), along with the estimated frequency of LS in unselected tumours (Table 1) and frequency of MMR deficiency in tumours from LS gene carriers (Table 2), to extrapolate statistics for the accuracy of MMR deficiency testing to screen for LS. To do so we first calculated estimates of the true positive, false positive, true negative, and false negative rate in each tumour type, and used these to generate additional summary statistics (Table 4). As discussed above in Section 5.3, MMR deficiency testing of all Lynch-spectrum tumours to identify LS gene carriers had equivalent sensitivity to testing of CRCs and ECs. The yield (true positive rate) of LS diagnoses ranged from 0.8% in PaCs up to 27.0% in SNs, but the yield in CNS tumours was exceptionally low at 0.2% mainly due to the low predicted frequency of LS in these patients. Specificity estimates for Lynch-spectrum tumours were all greater than the estimated 74.9% specificity for testing of ECs. Negative predictive values (NPVs) were >99% for all tumour types excepting SNs. The positive predictive value (PPV) varied greatly between Lynch-spectrum tumour types, from 5.1% in GCs up to 63.9% in SNs, and the majority had a PPV equal to or greater than the 20.8% estimated for CRCs. ECs had one of the lowest PPVs at 8.2%, though it should be noted that these estimates do not include *MLH1* promoter methylation testing to decrease the false positive rate. Interestingly, the PPV of MMR deficiency testing in PrCs for LS screening was very high at 78.8%, and the estimated yield was similar to some Lynch-spectrum tumours at 0.8%, again supporting screening for LS in PrC patients. MMR deficiency testing of BCs to screen for LS had a low yield at 0.2%, suggesting that this is not a useful intervention. Similarly, due to low PPV (9.1%) and yield (0.1%) estimates, MMR deficiency testing of non-Lynch-spectrum tumours was shown to be a poor screen for LS.

Together, these extrapolated statistics suggest that MMR deficiency testing of Lynch-spectrum tumours, excepting CNS tumours and SNs, to screen for LS would have equivalent accuracy to MMR deficiency testing of CRCs and ECs. However, these estimates must be interpreted with caution due to the wide confidence intervals in some of the underlying data for some tumour types. SNs were exceptional as immediate germline MMR gene testing is supported by the high frequency of LS in these patients and the lower NPV of MMR deficiency testing. CNS tumours are discussed in more detail in Section 5.5. LS screening by MMR deficiency testing of PrCs may also be a viable strategy given the enrichment of LS gene carriers among PrC patients and the high PPV of screening. The evidence does not support screening of BC or non-Lynch-spectrum tumour patients for LS. The false positive rate of LS screening by MMR deficiency testing of Lynch-spectrum tumours may also be improved by *MLH1* methylation testing, as it is for CRCs and ECs. For example, 92.9% (13/14) of sporadic MMR-deficient GCs had *MLH1* promoter methylation [236], whereas 0.0% (0/14) of MLH1-deficient LS CNS tumours and UCs had *MLH1* promoter methylation [28]. In addition, *MLH1* promoter methylation testing has been shown to increase the specificity of LS screening in OCs [237] and in SBCs [171]. However, additional research into the efficacy of *MLH1* promoter methylation testing in each tumour type is needed, as are analyses of cost-effectiveness before expansion of screening guidance to include MMR deficiency testing of all Lynch-spectrum tumours is considered.

### 5.5. MSI Analysis of CNS Tumours Has a Low Sensitivity for MMR Deficiency

Of the Lynch-spectrum tumours, CNS tumours had the lowest frequency of LS (0.3%), the lowest rate of MMR deficiency in LS gene carriers (73.9%), and the lowest rate of MMR deficiency in the unselected population (1.0%). This may, in part, be due to the limited number of independent studies of CNS tumours included in this review, and a lack of tumour subtyping in some of these, allowing for unintentional bias in frequency estimates. Therefore, any conclusions regarding LS screening in this Lynch-spectrum tumour are uncertain, and additional studies are needed. However, one interesting aspect is that the frequency of MMR deficiency in CNS tumours may depend on the detection method used. For example, Bonneville et al. [208] found that only 0.3% (3/909; 95% CI: 0.1–1.0%) of glioblastoma were MMR deficient using MSI analysis, but Tepeoglu et al. [233] found, in contrast, that 9.9% (7/71; 95% CI: 4.1–19.3%) were MMR deficient using IHC. Studies of glioblastoma diagnosed in patients with constitutional mismatch repair deficiency (CMMRD), a paediatric cancer predisposition syndrome caused by germline pathogenic variants in both alleles of an MMR gene [238], have previously shown that increased MSI could not be detected despite loss of MMR protein expression in the tumour or detection of MSI-H in synchronous adenocarcinomas [239,240,241]. It has been proposed that this is due to common loss of polymerase proof reading in parallel to MMR deficiency, leading to a complete loss of replication-associated repair, rapid tumour progression, and insufficient cell divisions or clonality to acquire detectable microsatellite variants using FLA or NGS [242]. Similarly, Gylling et al. [28] showed that FLA was unable to detect increased MSI in CNS tumours from LS gene carriers, whilst small pool PCR-based MSI analysis and IHC showed the tumours to be MMR deficient. This dissociation of MMR deficiency and MSI has also been observed in sporadic glioblastomas [243]. Therefore, should LS screening in CNS tumours be shown to be useful in the future, it is likely that MMR deficiency testing of CNS tumours will require IHC rather than MSI analysis.

### 5.6. MMR Deficiency Testing of Colorectal Adenomas in High-Risk Patients Could Be Used to Screen for LS

Recent meta-analyses have found that 69.5% (614/883; 95% CI: 66.4–72.6) to 76.7% (491/640; 95% CI: 73.3–79.9) of LS colorectal adenomas are MMR deficient [67,244], much higher than the 2.8% (60/2119; 95% CI: 2.2–3.6) of colorectal adenomas from the general population [244]. Zhu et al. [245] assessed the utility of MMR deficiency testing of colorectal adenomas to screen for LS using IHC. They detected MMR deficiency in 1.2% (6/508) of unselected colorectal adenomas, and germline MMR gene testing of these patients resulted in an LS diagnosis in 0.6% (3/508, 95% CI: 0.1–1.7%) of the cohort. Whilst this yield is not significantly greater than the expected population frequency of 0.3% [7], all of these diagnoses were made in patients aged <50 years, suggesting that MMR deficiency testing of adenomas from young patients may enrich for LS gene carriers [245]. In agreement, an independent study found that 3.2% (4/125) of advanced adenomas from patients aged <50 years were MMR deficient, leading to an LS diagnosis in 2.4% (3/125) of the cohort [246]. Similarly, 5.3% (3/57) of colorectal adenomas from familial cancer clinic patients were MMR deficient, and germline MMR gene testing of these patients showed that 3.5% (2/57) of the cohort had LS [247]. These yields of LS diagnoses from molecular screening of selected colorectal adenomas are equivalent to universal molecular screening of CRCs, suggesting that young or high-risk patients with colorectal adenomas could be screened for LS following current guidance for CRCs.

## 6. Non-Neoplastic Features Can Identify LS Gene Carriers Prior to Tumour Onset

Identification of LS from non-neoplastic features would allow LS individuals and families who have not presented with CRC, or other cancers, to be identified and offered management before tumour onset. One non-neoplastic feature that could be used to identify LS patients is the presence of MMR-deficient crypt foci (MMR-DCF) in the normal colorectal mucosa. MMR-DCF appear to be histologically normal by haematoxylin and eosin staining but have lost expression of the germline-affected MMR gene upon IHC examination [248]. Using biopsies of normal colorectal mucosa, the presence of MMR-DCF has been used to identify LS gene carriers with 69.7% (23/33) sensitivity and 100% (0/12) specificity. The LS gene carriers included individuals carrying a pathogenic variant from each of the four MMR genes and *EPCAM*, as well as individuals with and without a history of CRC or other cancers. Four carriers of MMR VUS were also analysed, and two were found to have MMR-DCF indicative of LS, which resolved their uncertain genetic diagnosis [249]. However, MMR-DCF are rare, with approximately one MMR-DCF per cm^2^ of LS mucosa analysed [248], equivalent to one MMR-DCF per 10,000 crypts [250], making this an impractical approach.

MMR-DCF show that MMR deficiency can strike in the non-neoplastic tissues of LS gene carriers, irrespective of their clinical history. Furthermore, expression knockdown experiments in human cell lines have shown that halving the expression of each of the four MMR genes associated with LS creates a significant reduction in repair capacity using an in vitro MMR assay [251,252]. It is hypothesised that this reduced MMR associated with 50% gene expression may also be found in vivo in the non-neoplastic cells of LS gene carriers [251,252], and the method is being developed to use primary cell culture from skin biopsies into a functional LS carrier assay: DiagMMR (LS CancerDiag). However, the need for skin biopsy and primary cell culture may limit widespread adoption of this approach. An alternative carrier assay could analyse the natural history of MMR deficiency in the normal soma of LS gene carriers through MSI analysis. For example, increased MSI is detectable in MMR-DCF, including frameshift mutations at coding monoNRs that are also present in MMR-deficient colorectal adenomas and adenocarcinomas [248,253]. Small pool PCR of three diNRs has been used to detect very low frequency microsatellite variants at significantly higher frequencies in the non-neoplastic peripheral blood leukocytes (PBLs) and saliva buccal cells of LS gene carriers compared to non-LS controls [254,255]. This method has also been used to show that microsatellite variant frequencies in PBLs increase with age in the general population, in agreement with increased clonal haematopoiesis [256]. Another method, which combined PCR and bacterial cloning of amplicons for highly sensitive variant detection, also found an increase in monoNR variants in the PBLs of LS gene carriers compared to controls [257]. However, both small pool PCR and PCR combined with cloning are laborious or require specialist setups unsuited to diagnostic services. Recently, scalable NGS-based methods have been developed that detect low level MSI in the non-neoplastic PBLs of CMMRD patients [258,259], a signal that is not detectable by traditional FLA [260]. Both NGS-based methods were able to perfectly separate CMMRD from control PBLs, including CMMRD due to hypomorphic variants in *PMS2*, but could not differentiate LS gene carrier from control PBLs [258,259]. Together, these results suggest that only the most sensitive of methods can detect increased MSI in the non-neoplastic tissues of LS gene carriers, but the current methods available are unsuited to high throughput LS screening.

## 7. MMR Deficiency Testing to Inform the Use of Immunotherapy Will Increase LS Detection

The release of frameshift peptides by MMR-deficient tumour cells makes them highly “visible” to a patient’s immune system. Hence, MMR-deficient cancers frequently evolve immune evasion mechanisms, including expression of immune checkpoint ligands on the tumour cell surface to repress immune cell attack [261]. In 2017, Le et al. reported durable responses to the anti-PD-1 immune checkpoint antibody, pembrolizumab, in advanced MMR-deficient cancers irrespective of tumour site. Subsequently, pembrolizumab is the first anti-neoplastic agent to be given a site agnostic licence, dependent only on the presence of tumour MMR deficiency [3]. Recently, pembrolizumab has also been shown to be a more effective and a safer first line therapy for advanced MMR-deficient CRC than chemotherapy [262]. Another drug in this class, nivolumab, has been developed and shown to work against melanoma in combination with another monoclonal antibody, ipilimumab, which deregulate immune checkpoints by blocking CTLA-4 signalling [263]. All have a significant adverse event profile, but the therapeutic benefit is likely to lead to their rapid uptake, and, in turn, a major impetus towards classification of MMR status in a range of tumours. This will inevitably lead to increased LS detection [70], and will facilitate germline MMR gene testing of select patients whilst still under acute care, allowing identified LS cases to benefit from not only immune checkpoint blockade therapy [3], but other acute clinical interventions such as more extensive surgery to reduce risk of metachronous disease [264].

## 8. Conclusions

To identify LS gene carriers, current clinical guidelines recommend universal molecular screening of CRCs to select patients for germline MMR gene testing. This strategy is based on several decades of evidence that show MMR deficiency testing of CRCs to screen for LS is a highly sensitive and cost-effective clinical intervention, superior to screening by clinical or familial criteria. Barriers to guideline implementation include a lack of stake-holder education, a lack of dedicated resource (both financial and personnel), and logistical difficulties. Such barriers should be overcome with the impetus from diagnostic guidance established in the last few years, and as part of a genomics revolution in healthcare. However, stakeholders within LS screening pipelines should remain cognizant that current guidelines have limitations as the results of molecular tumour analyses and germline MMR gene testing are not always definitive: some LS tumours do not fit the archetype the guidelines are based on, and technological limitations or a lack of knowledge mean that causative germline (or mosaic) variants can be missed. New technologies, in particular NGS and long-read sequencing, may address some of these barriers and limitations, but these technologies have their own limitations or are still being developed.

Current guidance is focused on screening CRC patients and misses screening opportunities offered by the broader LS phenotype. Already there is substantial evidence supporting MMR deficiency testing of ECs to screen for LS. Here, we have shown that MMR deficiency testing of all Lynch-spectrum tumours could be a highly sensitive and specific strategy. The exceptionally high frequency of LS in sebaceous neoplasm patients suggests that immediate germline MMR gene testing would be appropriate in this rare skin tumour. Prostate cancers, though not of the classic Lynch spectrum, are also highlighted as potential tumours for MMR deficiency testing to screen for LS. Furthermore, MMR deficiency testing is likely to become standard of care in CRC, and possibly other, Lynch-spectrum tumours, to inform use of immunotherapy. This dual utility of MMR deficiency testing further supports its use for LS screening. MMR deficiency also occurs in the non-neoplastic tissues of LS gene carriers, and could allow the development of an LS gene carrier assay to facilitate detection prior to tumour onset and to complement uncertain genetic diagnoses. For now, there is strong evidence that LS screening by MMR deficiency testing of most, if not all, Lynch-spectrum neoplasia has the potential to significantly increase the detection of LS, allowing clinical management to avoid significant morbidity and mortality in this, one of the commonest “rare” genetic disorders.

## Figures and Tables

**Table 1:** Estimated frequency of Lynch syndrome (LS) among unselected patients of different Lynch-spectrum, and other, tumour types. Unselected means no selection of patient by clinical features, such as age or family history. However, some studies selected patients for germline MMR gene testing by tumour MMR deficiency testing.

Tumour Type	Frequency	Method	Reference
**Colorectal Cancer**	**426/14075 (3.0%, 2.7–3.3% [95% CI])**	**Total**	
	23/1066 (2.2%)	Germline MMR gene testing of patients with MMR-deficient tumours	Hampel et al., 2005 [159]
	18/500 (3.6%)	Germline MMR gene testing of patients with MMR-deficient tumours	Hampel et al., 2008 [160]
	312/10206 (3.1%)	Germline MMR gene testing of 2650 (26.0%) patients	Moreira et al., 2012 [8]
	33/1058 (3.1%)	Germline MMR gene testing of all patients	Yurgelun et al., 2017 [29]
	12/419 (2.9%)	Germline MMR gene testing of all patients	Hampel et al., 2018 [30]
	28/826 (3.4%)	Germline MMR gene testing of all patients	Latham et al., 2019 [70]
**Endometrial Cancer**	**188/7098 (2.7%, 2.3–3.1% [95% CI])**	**Total**	
	145/5882 (2.5%)	Meta-analysis of germline MMR gene testing with heterogeneity of patients and genes tested	Ryan et al., 2019 [9]
	6/111 (5.4%)	Germline MMR gene testing of all patients	Chao et al., 2019 [144]
	9/525 (1.7%)	Germline MMR gene testing of all patients	Latham et al., 2019 [70]
	18/239 (7.5%)	Germline MMR gene testing of patients with MMR-deficient and *MLH1* promoter methylation-negative tumours	Dondi et al., 2020 [161]
	10/341 (2.9%)	Germline MMR gene testing of patients with MMR-deficient and *MLH1* promoter methylation-negative tumours	Hampel et al., 2021 [145]
**Gastric Cancer**	**3/308 (1.0%, 0.2–2.8% [95% CI])**	**Total**	
	1/97 (1.0%)	Germline MMR gene testing of patients with MMR-deficient tumours	Christakis et al., 2019 [162]
	2/211 (0.9%)	Germline MMR gene testing of all patients	Latham et al., 2019 [70]
**Ovarian Cancer**	**51/3484 (1.4%, 1.1–1.9% [95% CI])**	**Total ^1^**	
	9–64/1893 (0.5–3.4%)	Known pathogenic-known and predicted pathogenic. Germline *MLH1*, *MSH2*, and *MSH6* gene testing of all patients	Pal et al., 2012 [163]
	4/656 (0.6%)	Germline *MLH1*, *MSH2*, and *MSH6* gene testing of all patients	Akbari et al., 2017 [164]
Majority serous	0/343 (0.0%)	Germline MMR gene testing of all patients	Latham et al., 2019 [70]
	6/502 (1.2%)	Germline MMR gene testing of 17/36 (47.2%) patients with MMR-deficient tumour	Leskela et al., 2020 [165]
Endometrioid or Clear Cell	4/90 (4.4%)	Germline MMR gene testing of 5/7 (71.4%) patients with MMR-deficient tumour	Vierkoetter et al., 2014 [158]
**Pancreatic Cancer**	**41/3608 (1.1%, 0.8–1.5% [95% CI])**	**Total**	
	4/290 (1.4%)	Germline MMR gene testing of all patients	Grant et al., 2015 [166]
	5/249 (2.0%)	Germline MMR gene testing of all patients	Connor et al., 2017 [167]
	9/833 (1.1%)	Germline MMR gene testing of all patients	Hu et al., 2018 [168]
	1/199 (0.5%)	Germline MMR gene testing of patients with MMR-deficient tumours	Christakis et al., 2019 [162]
	8/824 (1.0%)	Germline MMR gene testing of all patients	Latham et al., 2019 [70]
	14/1213 (1.2%)	Germline MMR gene testing of 519/1213 (42.8%) patients	Grant et al., 2020 [169]
**Small Bowel Cancer**	**34/666 (5.1%, 3.6–7.1% [95% CI])**	**Total**	
	8/195 (4.1%)	Germline MMR gene testing of patients with MMR-deficient tumours	Jun et al., 2017 [170]
	4/29 (13.8%)	Germline MMR gene testing of patients with MMR-deficient tumours	Christakis et al., 2019 [162]
	2/57 (3.5%)	Germline MMR gene testing of all patients	Latham et al., 2019 [70]
	20/385 (5.2%)	Germline MMR gene testing of patients with MMR-deficient and *MLH1* promoter methylation-negative tumours	Suerink et al., 2020 [171]
**Urothelial Cancer**	**40/1589 (2.5%, 1.8–3.4% [95% CI])**	**Total**	
	13/551 (2.4%)	Germline MMR gene testing of all patients	Latham et al., 2019 [70]
	12/586 (2.0%)	Germline MMR gene testing of all patients	Carlo et al., 2020 [172]
Upper tract urothelial	6/194 (3.1%)	Germline MMR gene testing of patients with MMR-deficient tumours	Harper et al., 2016 [173]
Upper tract urothelial	7/115 (6.1%)	Germline MMR gene testing of patients with MMR-deficient tumours	Metcalfe et al., 2018 [174]
Upper tract urothelial	2/143 (1.4%)	Germline MMR gene testing of 2/7 (28.6%) patients with MMR-deficient tumours	Urakami et al., 2018 [175]
**Biliary Tract Cancer**	**6/250 (2.4%, 0.9–5.2% [95% CI])**	**Total**	
Ampulla of Vater	0/11 (0.0%)	Germline MMR gene testing of patients with MMR-deficient tumours	Christakis et al., 2019 [162]
Bile duct	0/60 (0.0%)	Germline MMR gene testing of patients with MMR-deficient tumours	Christakis et al., 2019 [162]
Gall bladder	0/19 (0.0%)	Germline MMR gene testing of patients with MMR-deficient tumours	Christakis et al., 2019 [162]
Periampullary	6/160 (3.8%)	Germline MMR gene testing of patients with MMR-deficient tumours	Gingras et al., 2015 [176]
**Sebaceous Neoplasms**	**87/261 (33.3%, 27.6–39.4% [95% CI])**	**Total**	
	11/24 (45.8%)	Germline MMR gene testing of all patients	Dandapani et al., 2011 [177]
	25/86 (29.1%)	Germline MMR gene testing of 58/86 (67.4%) patients	Everett et al., 2014 [178]
	40/89 (44.9%)	Germline MMR gene testing of all patients	Roberts et al., 2014 [179]
	11/62 (17.7%)	9/62 (14.5%) known genetic diagnosis, germline MMR gene testing of 10/53 (18.9%) remaining patients	Schon et al., 2018 [180]
**CNS Tumour**	**3/923 (0.3%, 0.1–0.9% [95% CI])**	**Total**	
	3/923 (0.3%)	Germline MMR gene testing of all patients	Latham et al., 2019 [70]
**Breast Cancer**	**11/3011 (0.4%, 0.2–0.7% [95% CI])**	**Total**	
	4/640 (0.6%)	Germline MMR gene testing of all patients	Davies et al., 2017 [181]
	7/2371 (0.3%)	Germline MMR gene testing of all patients	Latham et al., 2019 [70]
**Prostate Cancer**	**68/5831 (1.2%, 0.9–1.5% [95% CI])**	**Total**	
	7/1048 (0.7%)	Germline MMR gene testing of all patients	Latham et al., 2019 [70]
	58/3607 (1.6%)	Germline MMR gene testing of all patients	Nicolosi et al., 2019 [182]
	3/1176 (0.3%)	Germline *MSH2* gene testing of 12/14 (85.7%) patients with MSH2-deficient tumours	Guedes et al., 2020 [183]
**Non-Lynch-Spectrum Cancer**	**32/9116 (0.4%, 0.2–0.5% [95% CI])**	**Total**	
	24/7366 (0.3%)	Germline MMR gene testing of all patients	Latham et al., 2019 [70]
Lung	1/341 (0.3%)	Germline MMR gene testing of patients with MMR-deficient tumours	Takamochi et al., 2017 [184]
Lung	6/1179 (0.5%)	Germline MMR gene testing of all patients	Sun et al., 2019 [185]
Oesophageal	1/230 (0.4%)	Germline MMR gene testing of patients with MMR-deficient tumours	Christakis et al., 2019 [162]

^1^ Totals including studies with methodological uncertainty use the mean of the range of uncertainty, rounded to the nearest integer.

**Table 2:** Estimated frequency of mismatch repair (MMR) deficiency in different tumour types from Lynch syndrome (LS) gene carriers. Studies were only included if the LS diagnosis was made independent of tumour MMR status.

Tumour Type	Frequency	Method	Reference
**Colorectal Cancer**	**135/140 (96.4%, 91.9–98.8% [95% CI])**	**Total ^1^**	
	46/48 (95.8%)/35/35 (100.0%)	MSI by FLA of 2 monoNRs and 3 diNRs (Bethesda panel)/IHC of MLH1, MSH2, and MSH6	Gylling et al., 2008 [28]
	28/29 (96.6%)	MSI analysis by unspecified method and/or IHC of unspecified panel	Yurgelun et al., 2017 [29]
	54/58 (93.1%)/53/58 (91.4%)	MSI by FLA of 5 monoNRs (Promega), and MSI by NGS (mSINGS)/IHC of all 4 MMR proteins	Hampel et al., 2018 [30]
	18/18 (100.0%)	MSI by FLA of 2 monoNRs, and/or IHC of all 4 MMR proteins	Porkka et al., 2020 [31]
**Endometrial Cancer**	**79/98 (80.6%, 71.4–87.9% [95% CI])**	**Total ^1^**	
	8/8 (100.0%)/9/9 (100.0%)	MSI by FLA of 3 monoNRs and 3 diNRs (adapted Bethesda panel)/IHC of MLH1, MSH2, and MSH6	Lu et al., 2007 [188]
	38/60 (63.3%)/42/42 (100.0%)	MSI by FLA of 2 monoNRs and 3 diNRs (Bethesda panel)/IHC of MLH1, MSH2, and MSH6	Gylling et al., 2008 [28]
	19/21 (90.5%)	MSI (unspecified) and IHC of all 4 MMR proteins	Ring et al., 2016 [189]
	5/12 (41.7%)/10/13 (76.9%)	MSI by FLA of 3 monoNRs and 3 diNRs (adapted Bethesda panel)/IHC of all 4 MMR protein	Rubio et al., 2016 [143]
	4/4 (100.0%)/4/6 (66.7%)	MSI by FLA of 5 monoNRs (Sinomdgene Co. Ltd., Beijing China)/IHC of all 4 MMR proteins	Chao et al., 2019 [144]
**Gastric Cancer**	**35/39 (89.6%, 75.8–97.1% [95% CI])**	**Total ^1,2^**	
	7-15/15 (46.7–100.0%)	MSI-H-possible MSI-H due to inconclusive results. MSI by FLA of 7 diNRs	Aarnio et al., 1997 [186]
	13/13 (100.0%)/10/10 (100.0%)	MSI by FLA of 2 monoNRs and 3 diNRs (Bethesda panel)/IHC of MLH1, MSH2, and MSH6	Gylling et al., 2008 [28]
	4/4 (100.0%)/4/4 (100.0%)	MSI by FLA of 5 monoNRs (Promega) or 2 monoNRs and 3 diNRs (Bethesda panel)/IHC of all 4 MMR proteins	Fornasarig et al., 2018 [190]
	8/8 (100.0%)	IHC of all 4 MMR proteins	Saita et al., 2018 [191]
**Ovarian Cancer**	**38/40 (93.8%, 83.1–99.4% [95% CI])**	**Total ^1^**	
	19/20 (95.0%)/16/20 (80.0%)	MSI by FLA of 2 monoNRs/IHC of all 4 MMR proteins	Niskakoski et al., 2013 [192]
	4/4 (100.0%)	MSI by FLA of 2 monoNRs and 3 diNRs (Bethesda panel)	Akbari et al., 2017 [164]
	16/16 (100.0%)	MSI by FLA of 2 monoNRs, and/or IHC of all 4 MMR proteins	Porkka et al., 2020 [31]
**Pancreatic Cancer**	**22/29 (75.9%, 56.5–89.7% [95% CI])**	**Total**	
	3/3 (100.0%)	MSI by FLA of 2 monoNRs and 3 diNRs (Bethesda panel)	Yamamoto et al., 2001 [187]
	2/2 (100.0%)	IHC of all 4 MMR proteins	Grant et al., 2015 [166]
	3/5 (60.0%)	Mutational signatures (Alexandrov et al., 2013), confirmed by MSI by FLA of 5 monoNRs (Promega) and IHC of all 4 MMR proteins	Connor et al., 2017 [167]
	7/9 (77.8%)	MSI by NGS (MSIsensor), MSI by FLA of 5 monoNRs, and/or IHC of all 4 MMR proteins	Hu et al., 2018 [168]
	7/10 (70.0%)	IHC of all 4 MMR proteins, 10/14 (71.4%) LS tumours tested	Grant et al., 2020 [169]
**Small Bowel Cancer**	**22/23 (95.6%, 78.1–100.0% [95% CI])**	**Total ^1^**	
	21/21 (100.0%)/16/18 (88.9%)	MSI by FLA of 3 monoNRs and 3 diNRs (adapted Bethesda panel)/IHC of MLH1, MSH2, and MSH6	Schulmann et al., 2005 [137]
	2/2 (100.0%)	IHC of all 4 MMR proteins	Roth et al., 2016 [193]
Jejunal	1/1 (100.0%)/1/1 (100.0%)	MSI method not specified/IHC of all 4 MMR proteins	McIlvried et al., 2011 [194]
**Urothelial Cancer**	**26/30 (85.0%, 69.3–96.2% [95% CI])**	**Total ^1^**	
Bladder urothelial	3/5 (60.0%)/4/4 (100.0%)	MSI by FLA of 2 monoNRs and 3 diNRs (Bethesda panel)/IHC of MLH1, MSH2, and MSH6	Gylling et al., 2008 [28]
Bladder urothelial	6/7 (85.7%)/14/17 (82.4%)	MSI by FLA of 3 monoNRs and 3 diNRs (adapted Bethesda panel)/IHC of all 4 MMR proteins	van der Post et al., 2010 [138]
Bladder urothelial	5/5 (100.0%)	IHC of all 4 MMR proteins	Saita et al., 2018 [191]
Upper tract urothelial	6/9 (66.7%)/8/8 (100.0%)	MSI by FLA of 2 monoNRs and 3 diNRs (Bethesda panel)/IHC of MLH1, MSH2, and MSH6	Gylling et al., 2008 [28]
**Biliary Tract Cancer**	**6/6 (100.0%, 54.1–100.0% [95% CI])**	**Total**	
	5/5 (100.0%)	IHC of all 4 MMR proteins, 5/11 (45.5%) LS tumours tested	Cloyd et al., 2017 [195]
Ampulla of Vater	1/1 (100.0%)	IHC of all 4 MMR proteins	Roth et al., 2016 [193]
**Sebaceous Neoplasms**	**47/58 (81.0%, 68.6–90.1% [95% CI])**	**Total**	
	13/16 (81.3%)	IHC of all 4 MMR proteins	Everett et al., 2014 [178]
	27/34 (79.4%)	IHC of all 4 MMR proteins, numbers represent patients with all tumours showing MMR protein loss	Roberts et al., 2014 [179]
	7/8 (87.5%)	IHC of all 4 MMR proteins	Roth et al., 2016 [193]
**CNS Tumour**	**13/18 (73.9%, 46.5–90.3% [95% CI])**	**Total ^1^**	
	0/7 (0.0%) / 4/4 (100.0%)/3/4 (75.0%)	MSI by FLA of 2 monoNRs and 3 diNRs (Bethesda panel)/MSI by small-pool PCR of 2 diNRs/IHC of MLH1, MSH2, and MSH6	Gylling et al., 2008 [28]
	8/10 (80.0%)	IHC of all 4 MMR proteins	Therkildsen et al., 2015 [196]
Glioblastoma	1/1 (100.0%)	IHC of all 4 MMR proteins	Park et al., 2009 [197]
Glioblastoma	1/1 (100.0%)	IHC of all 4 MMR proteins	Lehrer et al., 2015 [198]
Glioblastoma	1/1 (100.0%)	IHC of all 4 MMR proteins	Kurtzman et al., 2015 [199]
**Breast Cancer**	**43/84 (50.9%, 40.0–62.3% [95% CI])**	**Total ^1^**	
	18/35 (51.4%)	IHC of all 4 MMR proteins	Walsh et al., 2010 [200]
	8/23 (34.8%)/13/20 (65.0%)	MSI by FLA of 2 monoNRs and 3 diNRs (Bethesda panel)/IHC of MLH1, MSH2, and MSH6	Lotsari et al., 2012 [201]
	2/4 (50.0%)	Mutational signatures (Alexandrov et al., 2013), confirmed by IHC of all 4 MMR proteins	Davies et al., 2017 [181]
	1/3 (33.3%)	IHC of all 4 MMR proteins	Saita et al., 2018 [191]
	11/20 (55.0%)	MSI by FLA of 2 monoNRs, and/or IHC of all 4 MMR proteins	Porkka et al., 2020 [31]
**Prostate Cancer**	**47/72 (65.7%, 53.1–76.1% [95% CI])**	**Total ^1^**	
	32/44 (72.7%)	Meta-analysis of studies using IHC	Ryan et al., 2014 [202]
	2/16 (12.5%)/11/16 (68.8%)	MSI by FLA of 5 monoNRs (Promega)/IHC of all 4 MMR proteins	Dominguez-Valentin et al., 2016 [203]
	1/1 (100.0%)	IHC of all 4 MMR proteins	Saita et al., 2018 [191]
	7/11 (63.6%)/8/10 (80.0%)	MSI by FLA of 5 monoNRs (Promega)/IHC of all 4 MMR proteins	Antonarakis et al., 2019 [204]
**Non-Lynch-Spectrum Cancer**	**2/11 (18.2%, 2.3–51.8% [95% CI])**	**Total**	
	0/3 (0.0%)	IHC of all 4 MMR proteins	Saita et al., 2018 [191]
Lung	0/6 (0.0%)	MSI by NGS (MSIsensor), MSI by FLA of 5 monoNRs, and/or IHC of all 4 MMR proteins	Sun et al., 2019 [185]
Oesophageal	1/1 (100.0%)	IHC of MSH2 and MSH6	Sweetser et al., 2013 [205]
Oesophageal	1/1 (100.0%)	IHC of all 4 MMR proteins	Sasaki et al., 2019 [206]

^1^ Totals use mean positive and mean N where a study used multiple methods to determine frequency, rounded to the nearest integer, to account for varying sensitivity and specificity of methods.^2^ Totals including studies with methodological uncertainty use the mean of the range of uncertainty, rounded to the nearest integer.

**Table 3:** Estimated frequency of mismatch repair (MMR) deficiency among unselected tumours of different Lynch-spectrum, and other, tumour types. Unselected means no selection of tumours by the patients’ clinical features, such as age or family history.

Tumour Type	Frequency	Method	Reference
**Colorectal Cancer**	**1823/13093 (13.9%, 13.3–14.5% [95% CI])**	**Total ^1^**	
	63/509 (12.4%)	MSI by Southern blot of 7 diNRs (custom panel), or by FLA of 16 diNRs (custom panel)	Aaltonen et al., 1998 [207]
	135/1066 (12.7%)	MSI by FLA of 2 monoNRs and 3 diNRs (Bethesda panel)	Hampel et al., 2005 [159]
	64/500 (12.8%)/71/483 (14.7%)	MSI by FLA of 2 monoNRs and 3 diNRs (Bethesda panel)/IHC of all 4 MMR proteins	Hampel et al., 2008 [160]
	1386/10019 (13.8%)	MSI by FLA of various microsatellite panels and/or IHC of various protein panels	Moreira et al., 2012 [8]
	77/419 (18.4%)	MSI by FLA of 5 monoNRs (Promega), and MSI by NGS (mSINGS)	Hampel et al., 2018 [30]
Colonic	85/431 (19.7%)	MSI by NGS (MANTIS)	Bonneville et al., 2017 [208]
Rectal	9/157 (5.7%)	MSI by NGS (MANTIS)	Bonneville et al., 2017 [208]
**Endometrial Cancer**	**1735/6522 (26.6%, 25.5–27.7% [95% CI])**	**Total ^1^**	
	170/542 (31.4%)	MSI by NGS (MANTIS)	Bonneville et al., 2017 [208]
	768/2890 (26.6%)/1948/7725 (25.2%)	Meta-analysis of studies using MSI analysis/meta-analysis of studies using IHC	Ryan et al., 2019 [9]
	12/83 (14.5%)/28/102 (27.5%)	MSI by FLA of 5 monoNRs (Sinomdgene Co. Ltd.)/IHC of all 4 MMR proteins	Chao et al., 2019 [144]
	96/239 (40.2%)	IHC of all 4 MMR proteins	Dondi et al., 2020 [161]
	91/341 (26.7%)	MSI by FLA of 5 monoNRs (Promega)/IHC of all 4 MMR proteins	Hampel et al., 2021 [145]
**Gastric Cancer**	**199/1123 (17.7%, 15.5–20.1% [95% CI])**	**Total**	
	5/56 (8.9%)	MSI by FLA of 2 monoNRs and 3 diNRs (Bethesda panel)	Toyota et al., 1999 [209]
	29/205 (14.1%)	MSI by FLA of 2 monoNRs and 3 diNRs (custom panel)	Yamamoto et al., 1999 [210]
	8/30 (26.7%)	MSI by 5 monoNRs and 3 diNRs (adapted Bethesda panel)	Leung et al., 2000 [211]
	64/295 (21.7%)	MSI by NGS (not specified)	Bass et al., 2014 [212]
	84/440 (19.1%)	MSI by NGS (MANTIS)	Bonneville et al., 2017 [208]
	9/97 (9.3%)	MSI by NGS (custom analysis)	Christakis et al., 2019 [162]
**Ovarian Cancer**	**289/2733 (10.6%, 9.4–11.8% [95% CI])**	**Total ^1^**	
	145/977 (14.8%)	Meta-analysis of studies using MSI analysis	Pal et al., 2008 [213]
	88/656 (13.4%)	MSI by FLA of 2 monoNRs and 3 diNRs (Bethesda panel)	Akbari et al., 2017 [164]
	36/502 (7.2%)	IHC of all 4 MMR proteins	Leskela et al., 2020 [165]
Endometrioid	7/71 (9.9%)/7/71 (9.9%)	MSI by FLA of 5 monoNRs (Promega)/IHC of all 4 MMR proteins	Aysal et al., 2012 [214]
Endometrioid or clear cell	7/90 (7.8%)	IHC of all 4 MMR proteins	Vierkoetter et al., 2014 [158]
Serous	6/437 (1.4%)	MSI by NGS (MANTIS)	Bonneville et al., 2017 [208]
**Pancreatic Cancer**	**224/8954 (2.5%, 2.2–2.8% [95% CI])**	**Total**	
	0/183 (0.0%)	MSI by NGS (MANTIS)	Bonneville et al., 2017 [208]
	4/249 (1.6%)	Mutational signatures (Alexandrov et al., 2013), confirmed by MSI by FLA of 5 monoNRs (Promega) and IHC of all 4 MMR proteins	Connor et al., 2017 [167]
	2/199 (1.0%)	MSI by NGS (custom analysis)	Christakis et al., 2019 [162]
	218/8323 (2.6%)	Meta-analysis of studies using MSI analysis and/or IHC	Luchini et al., 2020 [215]
**Small Bowel Cancer**	**136/704 (19.3%, 16.5–22.4% [95% CI])**	**Total**	
	16/89 (18.0%)	MSI by FLA of 4 monoNRs	Planck et al., 2003 [216]
	2/22 (9.1%)	MSI by FLA of 4 monoNRs and 6 diNRs (custom panel)	Potter et al., 2004 [217]
	14/61 (23.0%)	IHC of all 4 MMR proteins	Aparicio et al., 2013 [218]
	6/71 (8.5%)	MSI by 2 monoNRs and 3 diNRs (Bethesda panel)	Xia et al., 2017 [219]
	8/29 (27.6%)	MSI by NGS (custom analysis)	Christakis et al., 2019 [162]
	84/385 (21.8%)	IHC of all 4 MMR proteins	Suerink et al., 2020 [171]
Duodenal	6/47 (12.8%)	IHC of all 4 MMR proteins	Xue et al., 2017 [220]
**Urothelial Cancer**	**42/1200 (3.5%, 2.5–4.7% [95% CI])**	**Total ^1^**	
Bladder urothelial	0/54 (0.0%)/6/92 (6.5%)	MSI by FLA of 2 monoNRs and 3 diNRs (Bethesda panel)/IHC of all 4 MMR proteins	Giedl et al., 2014 [221]
Bladder urothelial	2/412 (0.5%)	MSI by NGS (MANTIS)	Bonneville et al., 2017 [208]
Bladder urothelial	1/160 (0.6%)	IHC of all 4 MMR proteins	Ju et al., 2018 [222]
Upper tract urothelial	10/194 (5.2%)	IHC of all 4 MMR proteins	Harper et al., 2016 [173]
Upper tract urothelial	10/117 (8.5%)	IHC of all 4 MMR proteins	Ju et al., 2018 [222]
Upper tract urothelial	5/87 (5.7%)/13/115 (11.3%)	MSI by FLA of 3 monoNRs, 3 diNRs, and 1 marker of ambiguous identity/IHC of all 4 MMR proteins	Metcalfe et al., 2018 [174]
Upper tract urothelial	7/143 (4.9%)	IHC of all 4 MMR proteins	Urakami et al., 2018 [175]
**Biliary Tract Cancer**	**68/993 (6.9%, 5.4–8.6% [95% CI])**	**Total ^1^**	
	4/126 (3.2%)	MSI by FLA of 2 monoNRs and 3 diNRs (Bethesda panel)	Rashid et al., 2002 [223]
Ampulla of Vater	8/53 (15.1%)	MSI by FLA of 8 diNRs (panel for LoH analysis)	Scarpa et al., 2000 [224]
Ampulla of Vater	5/53 (9.4%)	MSI by FLA of 5 monoNRs (panel of Suraweera et al., 2002)	Sessa et al., 2007 [225]
Ampulla of Vater	15/144 (10.4%)/11/139 (7.9%)	MSI by FLA of 3 monoNRs and 4 diNRs (adapted Bethesda panel)/IHC of MLH1, MSH2, and MSH6	Ruemmele et al., 2009 [226]
Ampulla of Vater	3/54 (5.6%)	IHC of all 4 MMR proteins	Agaram et al., 2010 [227]
Ampulla of Vater	0/11 (0.0%)	MSI by NGS (custom analysis)	Christakis et al., 2019 [162]
Bile duct	1/74 (1.4%)	MSI by NGS (MANTIS)	Bonneville et al., 2017 [208]
Bile duct	1/60 (1.7%)	MSI by NGS (custom analysis)	Christakis et al., 2019 [162]
Gall bladder	0/19 (0.0%)	MSI by NGS (custom analysis)	Christakis et al., 2019 [162]
Gall bladder	1/69 (1.4%)	MSI by FLA of 3 monoNRs (custom panel)	Goeppert et al., 2019 [228]
Periampullary	12/160 (7.5%)	MSI by NGS (custom analysis)	Gingras et al., 2015 [176]
Periampullary	20/172 (11.6%)	IHC of all 4 MMR proteins	Heby et al., 2018 [229]
**Sebaceous Neoplasms**	**584/1385 (42.2%, 39.5–44.8% [95% CI])**	**Total**	
	24/49 (49.0%)	IHC of all 4 MMR proteins	Mojtahed et al., 2011 [230]
	38/77 (49.4%)	IHC of all 4 MMR proteins	Everett et al., 2014 [178]
	71/74 (95.9%)	IHC of all 4 MMR proteins, numbers represent patients with at least one tumour showing MMR protein loss	Roberts et al., 2014 [179]
	143/216 (66.2%)	IHC of all 4 MMR proteins	Jessup et al., 2016 [231]
	26/50 (52.0%)	IHC of all 4 MMR proteins	Schon et al., 2018 [180]
	282/919 (30.7%)	IHC of all 4 MMR proteins	Walsh et al., 2018 [232]
**CNS Tumour**	**10/980 (1.0%, 0.5–1.9% [95% CI])**	**Total**	
Glioblastoma	1/396 (0.3%)	MSI by NGS (MANTIS)	Bonneville et al., 2017 [208]
Glioblastoma	7/71 (9.9%)	IHC of all 4 MMR proteins	Tepeoglu et al., 2019 [233]
Lower-grade glioma	2/513 (0.4%)	MSI by NGS (MANTIS)	Bonneville et al., 2017 [208]
**Breast Cancer**	**58/3319 (1.7%, 1.3–2.3% [95% CI])**	**Total**	
	16/1044 (1.5%)	MSI by NGS (MANTIS)	Bonneville et al., 2017 [208]
	11/640 (1.7%)	Mutational signatures (Alexandrov et al., 2013), confirmed by IHC of all 4 MMR proteins	Davies et al., 2017 [181]
	31/1635 (1.9%)	IHC of all 4 MMR proteins	Cheng et al., 2020 [234]
**Prostate Cancer**	**17/1674 (1.0%, 0.6–1.6% [95% CI])**	**Total**	
	3/498 (0.6%)	MSI by NGS (MANTIS)	Bonneville et al., 2017 [208]
	14/1176 (1.2%)	IHC of MSH2	Guedes et al., 2020 [183]
**Non-Lynch-spectrum Cancer**	**55/7222 (0.8%, 0.6–1.0% [95% CI])**	**Total ^1^**	
	46/6012 (0.8%)	MSI by NGS (MANTIS)	Bonneville et al., 2017 [208]
Lung	1/341 (0.3%)	MSI by FLA of 5 monoNRs (Promega)	Takamochi et al., 2017 [184]
Oesophageal	2/362 (0.6%)/7/916 (0.8%)	MSI by FLA of 5 monoNRs (Promega)/IHC of various protein panels	Hewitt et al., 2018 [235]
Oesophageal	3/230 (1.3%)	MSI by NGS (custom analysis)	Christakis et al., 2019 [162]

^1^ Totals use mean positive and mean N where a study used multiple methods to determine frequency, rounded to the nearest integer, to account for varying sensitivity and specificity of methods.

**Table 4:** Extrapolated accuracy statistics of mismatch repair (MMR) deficiency testing of different tumour types to screen for Lynch syndrome.

Tumour Type	Frequency Estimates		MMR Deficiency Screen Results ^1^				Screening Accuracy			
	MMR Deficiency ^2^	Lynch Syndrome ^3^	True Positive	False Negative	True Negative	False Positive	Sensitivity ^4^	Specificity	PPV	NPV
Colorectal cancer	13.9%	3.0%	2.9%	0.1%	86.0%	11.0%	96.4%	88.7%	20.8%	99.9%
Endometrial cancer	26.6%	2.7%	2.2%	0.5%	72.9%	24.4%	80.6%	74.9%	8.2%	99.3%
Gastric cancer	17.7%	1.0%	0.9%	0.1%	82.2%	16.8%	89.6%	83.0%	5.1%	99.9%
Ovarian cancer	10.6%	1.4%	1.3%	0.1%	89.3%	9.3%	93.8%	90.6%	12.4%	99.9%
Pancreatic cancer	2.5%	1.1%	0.8%	0.3%	97.2%	1.7%	75.9%	98.3%	33.4%	99.7%
Small bowel cancer	19.3%	5.1%	4.9%	0.2%	80.5%	14.4%	95.6%	84.8%	25.3%	99.7%
Urothelial cancer	3.5%	2.5%	2.1%	0.4%	96.1%	1.4%	85.0%	98.6%	60.7%	99.6%
Biliary tract cancer	6.9%	2.4%	2.4%	0.0%	93.1%	4.5%	100.0%	95.4%	34.8%	100.0%
Sebaceous neoplasms	42.2%	33.3%	27.0%	6.3%	51.5%	15.2%	81.0%	77.2%	63.9%	89.1%
CNS tumour	1.0%	0.3%	0.2%	0.1%	98.9%	0.8%	73.9%	99.2%	22.2%	99.9%
Breast cancer	1.7%	0.4%	0.2%	0.2%	98.1%	1.5%	50.9%	98.5%	12.0%	99.8%
Prostate cancer	1.0%	1.2%	0.8%	0.4%	98.6%	0.2%	65.7%	99.8%	78.8%	99.6%
Non-Lynch-spectrum	0.8%	0.4%	0.1%	0.3%	98.9%	0.7%	18.2%	99.3%	9.1%	99.7%

^1^ The results of screening can be extrapolated from the frequency estimates in Table 1, Table 2 and Table 3. ^2^ The estimates of MMR deficiency frequency in unselected tumours are taken from Table 3. ^3^ The estimates of Lynch syndrome frequency in unselected tumours are taken from Table 1. ^4^ The estimates of MMR deficiency frequency in tumours from Lynch syndrome patients are taken from Table 2.

## Data Availability

The data presented in this study are publically available in the referenced articles.
